# AAA Syndrome, Case Report of a Rare Disease

**DOI:** 10.12669/pjms.336.13684

**Published:** 2017

**Authors:** S. Waqar H. Shah, Arshad K. Butt, K. Malik, Altaf Alam, Adnan Shahzad, Anwaar A. Khan

**Affiliations:** 1 Dr. S. Waqar H. Shah, MBBS. Department of Gastroenterology-Hepatology, Shaikh Zayed Medical Complex, Lahore, Pakistan; 2 Dr. Arshad K. Butt, FCPS. Department of Gastroenterology-Hepatology, Shaikh Zayed Medical Complex, Lahore, Pakistan; 3 Dr. K. Malik, FCPS. Department of Gastroenterology-Hepatology, Shaikh Zayed Medical Complex, Lahore, Pakistan; 4 Dr. Altaf Alam FRCP. Department of Gastroenterology-Hepatology, Shaikh Zayed Medical Complex, Lahore, Pakistan; 5 Adnan Shahzad, MBBS. Doctors Hospital and Medical Center, Lahore, Pakistan; 6 Dr. Anwaar A. Khan, M.D, MACP, FACG, FRCP. Doctors Hospital and Medical Center, Lahore, Pakistan

**Keywords:** Allgrove Syndrome, Alacrimia, Achalasia, ALADIN

## Abstract

Triple A (Allgrove) syndrome, an autosomal recessive disease is characterized by achalasia, alacrimia and ACTH-resistant adrenal failure with progressive neurological syndrome including central, peripheral and autonomic nervous system impairment, and mild mental retardation. The triple A syndrome gene, designated AAAS, localized on chromosome 12q 13 encodes for a 546 amino acid protein called ALADIN (Alacrimia-Achlasia-Adrenal Insufficiency and Neurologic disorder).

This report relates to two sisters, aged 8 and 12 years, who had vomiting, muscle weakness, alacrimia, excessive fatigue and dysphagia. Abdominal sonography, esophago-gastroduodenoscopy, barium swallow, esophageal manometry, CT scan abdomen and brain, biochemical profiles, as well as neurologic and ophthalmic evaluations were consistent with Allgrove’s syndrome. Management consisted of pneumatic balloon dilatation for achalasia and initiation of cortisone therapy with successful resolution of dysphagia and other symptoms.

## INTRODUCTION

The triple A (All grove) syndrome was first described in two pairs of unrelated siblings in 1978.^1^ Since then, a number of families have been reported, all of them displaying an autosomal recessive pattern of inheritance (Online Mendelian Inheritance in Man (OMIM) database accession number: 231550). Weber et al.^2^ localized the AAAS gene on chromosome 12q 13 and was cloned in 2000 by Tullio-Peletetal.^3^ and Hands chug et al in 2001.^4^ The AAA-S gene with high expression in brain, adrenals and GI tract mucosa encodes a 546 amino acid protein called ALADIN (Alacrimia-Achalasia-Adrenal Insufficiency Neurologic disorder). ALADIN protein belongs to the family of genes called WDR (WD repeat domain containing) which has important role in transnuclear movement of molecules.^4^-^6^ No morphological abnormalities have been reported in cells from patients with Allgrove syndrome, suggesting that mutations in AAA-S gene result in functional and not structural, abnormalities.^7^-^11^ Not all patients demonstrate mutations of the AAAS gene^4^,^7^,^10^,^12^ suggesting possible genetic heterogeneity.

Clinically affected patients present with achalasia, alacrimia and ACTH-resistant adrenal failure along with progressive neurological impairment with or without mild mental retardation.^8^-^11^ This is the first report of this rare syndrome from Pakistan.

## CASE REPORT

### Case 1

A 12 year-old girl BA, was referred to the GI Motility Lab, Department of Gastroenterology, at Shaikh Zayed Hospital, Lahore for evaluation of recurrent vomiting and hypoglycemic episodes and worsening of dysphagia. She was offspring of consanguineous marriage, born at 39 weeks of gestation. Developmental land marks and growth were reported to be normal, although she was often listless, had a poor appetite, and could not keep up with her friends at play, since one month of age, until she was 3½ years old. She was admitted to the hospital multiple times, for the treatment of upper and lower respiratory tract infections, vomiting and diarrhea. At the age of three years, her parents noticed lack of tears while crying. During some of these episodes, she received brief courses of hydrocortisone for chest infection. On physical examination, she was lean girl with slight pallor. Her blood pressure was 100/60 mm Hg with no postural drop. Her height and weight were at the 50^th^ and 75^th^ percentiles, respectively. There was no goiter. She had moderate hyper pigmentation of skin, gums, buccal mucosa, palmar creases, knuckles, and elbows. Rest of the general physical examination, and neurological examination was normal. Her performance at school was reported to be satisfactory.

Hemoglobin was 10.3 g/dL, hematocrit was 33%. Serum sodium was low at 128 mmol/L, potassium 3.7mmol/L, chloride 107 mmol/L, urea 19mg/dl, creatinine 1.2 mg/dl, and albumin was 3.8 gm/dl. Serum cortisol at 8 am was low at 2.6 ug/dl (normal 6-30). Post synacthen (ACTH stimulation test) was abnormal at 1.76 ug/dl, range (2.5-10.5). Abdominal x-ray and CT scan of the adrenal glands were normal.

**Fig.1:**
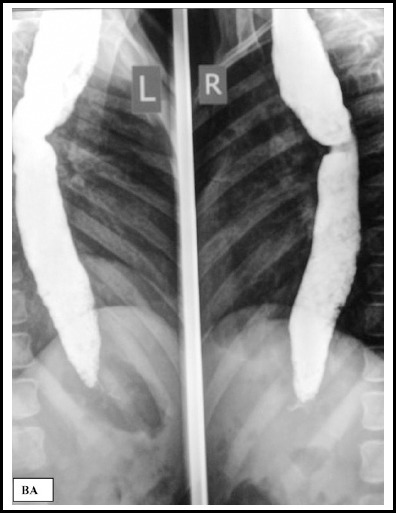
Pre dilatation barium swallow, dilated esophagus.

At 8 years of age, barium swallow was performed for evaluation of recurrent vomiting and mild dysphagia, and was reported normal. Repeat Barium swallow, at 12, showed moderately dilated esophagus with distal, smooth narrowing, typical of achalasia (Fig.1) subsequently, confirmed on esophageal manometry.

Ophthalmological examination, including Schirmer’s test was positive, wetting was 3 mm in right eye and 2.5 mm in left eye at five minutes, whereas, normal wetting at five minutes is >5mm. She had dry eyes and alacrima. CT scan of the brain, as well as electroencephalogram (EEG) were normal. She was found to have hypoadrenalism, showed substantial clinical improvement after starting on prednisolone. Her appetite improved, gained weight, became more active and playful, continues to take 12.5 mg prednisolone daily and artificial tears as maintenance.

**Fig.2:**
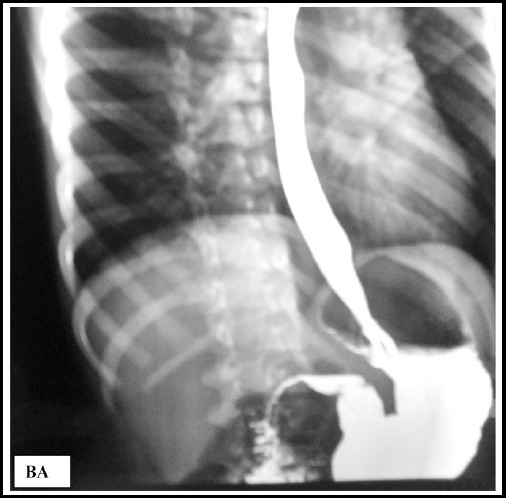
Post dilatation barium swallow, less dilated esophagus.

Based on presentation of achalasia, alacrima and adrenal insufficiency, diagnosis of triple A syndrome was made. She had pneumatic balloon dilatation with 30 mm diameter Microvasive balloon as reported earlier.^13^ Post dilatation barium swallow showed rapid entry of barium into the stomach, without any signs of perforation (Fig.2). On four month follow-up, she had relief of dysphagia, gained 2kg weight and her height increased from 46 cm to 51cm. Follow up manometry at four months, showed reduction in lower esophageal sphincter pressure from 21.5 mm Hg to 11.8 mm Hg.

### Case 2

Six year old BZ, younger sister of BA, was brought to the Pediatric outpatient department, for dark pigmentation of her lips, elbows and knuckles of hands, alacrima occasional vomiting. She had developed repeated episodes of vomiting three weeks prior to presentation. Her developmental mile stones were normal. On examination, she had generalized weakness, blood pressure was 80/40 mm Hg, height and weight were at the 20^th^ and the 30^th^ percentiles, respectively. There was mild, diffuse hyperpigmentation of skin, with more involvement of lips, gums, creases of the palms, buccal mucosa, and elbows. Neurological system was normal. Ophthalmological examination documented presence of alacrima and dry eyes, she had nasal speech.

**Fig.3:**
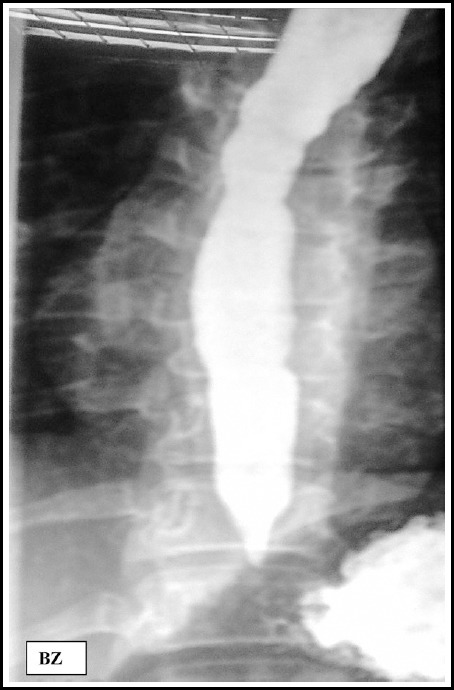
Pre dilatation barium swallow, dilated esophagus.

**Fig.4:**
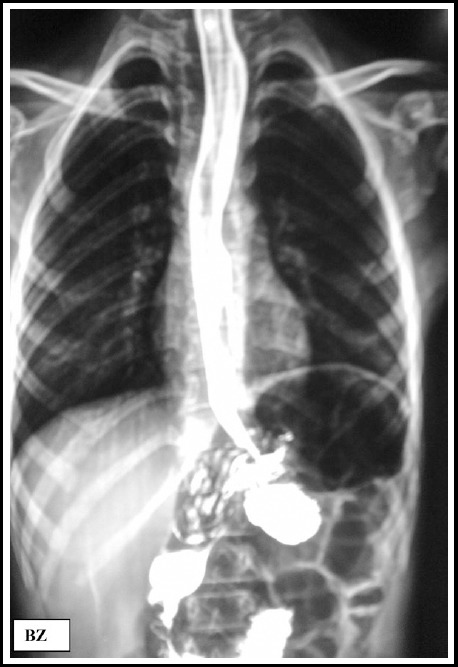
Post dilatation barium swallow, lesser diameter of esophagus.

Laboratory investigations revealed hemoglobin of 12.4g/dl, packed cell volume 36.8%, and total leukocyte count 130x10^9^ per liter. Platelet count was normal. Fasting serum glucose, serum electrolytes and renal function tests were normal. Serum cortisol at 8 am was low at 1.9 micrograms/dl (normal 5-28). Abdominal X-ray and CT scan of the adrenals were normal. Her chest X-ray showed bifid right 4^th^ rib, not reported earlier in this syndrome. Barium swallow revealed smooth tapering of lower esophagus and a delayed esophageal emptying (Fig.3). Aperistalsis on esophageal manometry confirmed diagnosis of achalasia. She had pneumatic balloon dilatation successfully, with Microvasive balloon, as described earlier.^13^ Post dilatation barium swallow (Fig.4) showed effective esophageal emptying with no sign of perforation. She is presently doing well on daily 10.0 mg prednisolone and artificial tears. She has gained weight and vomiting resolved. Follow up manometry after months, showed reduction in lower esophageal sphincter pressure from 20.2 mm Hg to 12.2 mm Hg.

## DISCUSSION

Allgrove’s syndrome is an autosomal recessive disorder with varied presentations.^14^,^15^ Recent studies have identified mutation in AAA syndrome of a candidate gene on chromosome 12 q 13 in these patients.^16^ Prpic et al (2003) demonstrated marked phenotypic variability in three patients with genetically confirmed triple A syndrome. Two patients had achalasia, alacrima and adrenocortical deficiency as well as neurologic and autonomic dysfunction, third patient had achalasia and neurologic dysfunction only. All patients were homozygous for mutations in the triple A syndrome gene.^16^ Age of onset of symptoms is variable, usually presenting during first decade of life with dysphagia and severe, occasionally fatal, hypoglycemia or hypotensive epidodes, related to adrenocortical insufficiency.^17^,^18^

Since the first reported case of AAA syndrome in 1978, over 100 cases presenting with the clinical features of esophageal achalasia, alacrimia, and adrenal insufficiency have been described.^19^ Alacrimia was the first sign to become evident in our patients, probably, it was already present at birth, as parents noted lack of tears on crying. Recurrent vomiting, poor appetite and failure to thrive were present at two years of age. These symptoms were probably due to achalasia, or early signs of adrenal insufficiency. In retrospect, adrenal insufficiency probably started at ages of three years, when hyper-pigmentation and gastro intestinal symptoms were first noted.

Recurrent chest infections and failure to thrive, required frequent hospital admissions in both patients. BA developed dysphagia, due to achalasia when she was 12 years old, whereas, her younger sister, BZ was diagnosed with achalasia at 8 years of age. The association of dry eyes, nasal speech, together with achalasia were important clinical features, in support of Allgrove’s syndrome. Lacrimal gland CT (orbital tomography) is helpful and biopsy obtained from lacrimal gland may show neuronal degeneration and depletion of secretory granules in the acinar cells.^18^,^20^

Etiology of neuropathy in Allgrove’s syndrome is obscure. At present, no explanation for the association of achalasia, alacrima and adrenal unresponsiveness to ACTH in the AAA syndrome is available. It has been suggested that the ACTH receptor gene could provide the link to explain the association of the three main features of this syndrome, since there is evidence that ACTH has some neuropathic effects.^21^,^22^

Barium swallow, esophagoscopy and manometry are needed to diagnose achalasia and should be considered besides the preliminary investigations for diagnosis.

In both patients, diagnosis of achalasia was established and both responded successfully to pneumatic balloon dilatation with subjective and objective improvement of dysphagia. Careful replacement of glucocorticoids was done and good control was achieved for adrenal insufficiency.

## CONCLUSION

Allgrove’s syndrome may be an under diagnosed disorder. High index of suspicion is needed when patients present with such complex symptoms at variable stages i.e. failure to thrive, dysphagia, crying without tears (alacrima), nasal speech and hypoglycemic seizures. It may be associated with neurological deficits. Diagnosis can be confirmed by esophageal manometry, ophthalmological assessment, biochemical studies and neurological evaluation. Effective, early management can result in near normal life span.
